# Determinants of brain network resilience after stroke

**DOI:** 10.1093/braincomms/fcaf218

**Published:** 2025-06-06

**Authors:** Elisabeth Dirren, Julian Klug, Cecilia Jarne, Diego Vidaurre, Emmanuel Carrera

**Affiliations:** Stroke Research Group, Department of Clinical Neurosciences, Geneva University Hospitals, Geneva 1205, Switzerland; Stroke Research Group, Department of Clinical Neurosciences, Geneva University Hospitals, Geneva 1205, Switzerland; Stroke Research Group, Department of Clinical Neurosciences, Geneva University Hospitals, Geneva 1205, Switzerland; Departamento de Ciencia y Tecnología, Universidad Nacional de Quilmes, Bernal B1876, Argentina; CONICET, Buenos Aires C1425FQB, Argentina; Department of Clinical Medicine, Center of Functionally Integrative Neuroscience, Aarhus University, Aarhus 8000, Denmark; Department of Clinical Medicine, Center of Functionally Integrative Neuroscience, Aarhus University, Aarhus 8000, Denmark; Stroke Research Group, Department of Clinical Neurosciences, Geneva University Hospitals, Geneva 1205, Switzerland

**Keywords:** connectivity, graph theory, virtual stroke, network robustness

## Abstract

Strokes lead to widespread network changes that are associated with functional deficits and subsequent recovery. Beyond function, we hypothesize in this study that stroke-induced reorganization of brain connectivity increases network resilience against recurrent events, as an adaptive mechanism to limit the functional consequences of a new lesion. We used a dataset of 75 first-time stroke patients with resting-state functional connectivity assessed at 3 time-points within 1 year of stroke to determine whether brain networks of stroke patients become more resistant to recurrent lesions. We defined resilience as the ability of brain networks to maintain their core integrative and modular properties following recurrent attacks. Because recurrent strokes are unpredictable in the clinical setting, we probed resilience by comparing whole-brain global efficiency and modularity before and after virtual strokes, which consisted in removing network nodes that overlapped with clinical stroke lesion masks. Global efficiency was chosen as a graph metric to represent network integration, whereas modularity was used as an indicator of the network’s modular structure. Both in terms of global efficiency and modularity, we observed greater resilience in patients than in controls. Resilience of global efficiency was greater in patients at 2 weeks and 3 months post primary stroke, whereas resilience of modularity was increased up to 1 year post stroke. We further considered architectural specificities of brain networks that may be associated with resilience, focusing on the distribution of nodal participation coefficient. We found that nodes with high participation coefficient in controls, so called hubs, had lower participation coefficient in stroke patients. Finally, we found that specific patient and primary lesion characteristics were associated with resilience. For instance, we observed increased resilience of global efficiency in younger patients and in those with high scores on the National Institutes of Health Stroke Scale, whereas resilience of modularity was associated with older age. Importantly, there was no association between resilience and primary stroke lesion size. Our results unveil potential connectivity mechanisms of network resilience after stroke that could be targeted by future therapeutic strategies to limit the impact of recurrent lesions.

## Teaser

Dirren *et al*. investigated whether the brain adapts to enhance resilience after a first stroke. By comparing network properties before and after simulating recurrent strokes, they found that patients’ brain networks showed greater resilience, being less affected by recurrent virtual lesions compared to controls.

## Graphical Abstract

**Graphical Abstract:**
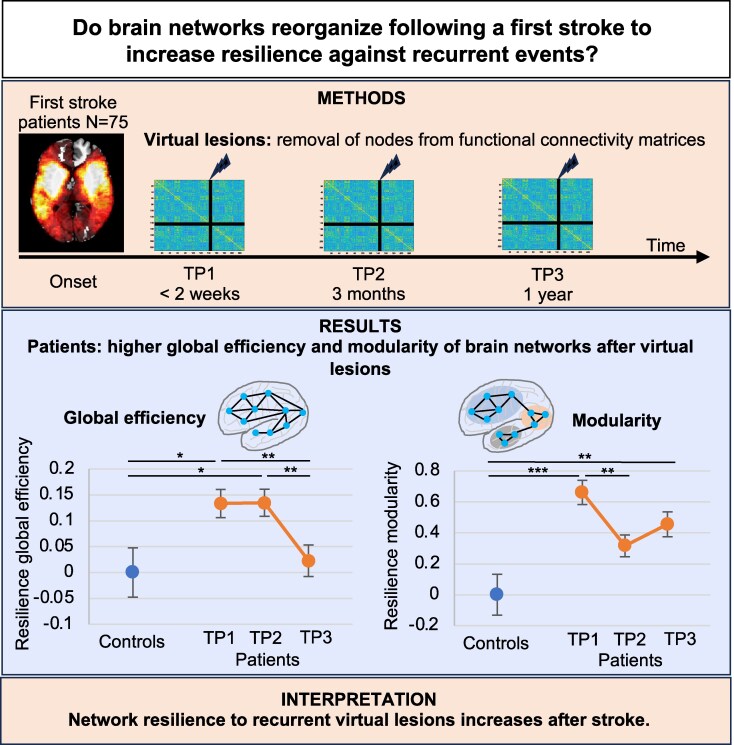

## Introduction

Stroke is a leading cause of morbidity and mortality worldwide, with over 12 million incident cases reported in 2019.^1-3^ Despite optimized secondary prevention, stroke recurrence is frequent, ranging from 2 to 8% at 3 months and 5 to 20% at 1 year.^4-8^ Surprisingly, the functional burden of recurrent focal lesions might not simply add up on previous deficits.^9^ For instance, patients who survive a recurrent stroke may recover to the same extent and as fast as patients with a first stroke.^10,11^ Brain resilience to recurrent insults might therefore build up following a first event, as an adaptative mechanism to limit the functional consequences of a new attack.^12^ Enhancing resilience for secondary functional prevention could have a huge impact on global stroke burden.

Human brains display a core anatomical and functional architecture that allows information processing throughout widespread cortical and subcortical areas, while maintaining specialization of function in dedicated regions.^13,14^ One way to characterize high-level features of this architecture is using graph theory, which conceives the system in terms of nodes and connections between nodes. Of particular interest, global efficiency and modularity are graph metrics that measure the capacity of a network to support integrative and specialized tasks, respectively.^13-15^ Global efficiency has been associated with working memory and intellectual performance,^16,17^ whereas modularity has been associated with long-term memory and motor learning.^18,19^ Both metrics are impacted by stroke.^20-23^ Computational modelling of the brain’s response to targeted attacks has shown not only widespread network modifications but also remarkable resilience, with the preservation of key network features of functional integration and specialization, despite the removal of central nodes.^15,24-27^ In these studies, attacks were modelled by removing random or targeted nodes of the network architecture, rather than by removing nodes related to clinical lesions.

Studying brain resilience in stroke patients is challenging due to the unpredictable timing and location of recurrent strokes. Simulating the impact of virtual lesions on brain networks offers a valuable approach to circumvent this limitation. Operationally, we simulated virtual lesions by removing nodes that overlapped with clinical stroke lesion masks.^28,29^ We have recently used this strategy in a population of stroke patients.^29^ However, this study was limited to a small and highly selected population with first-ever ischaemic strokes restricted to the primary motor cortex. Therefore, it remains unknown whether resilience is a generalized pathophysiological process and a clinically relevant mechanism. Similarly, patient and primary lesion characteristics that may impact network resilience are unknown.

**Figure 1:**
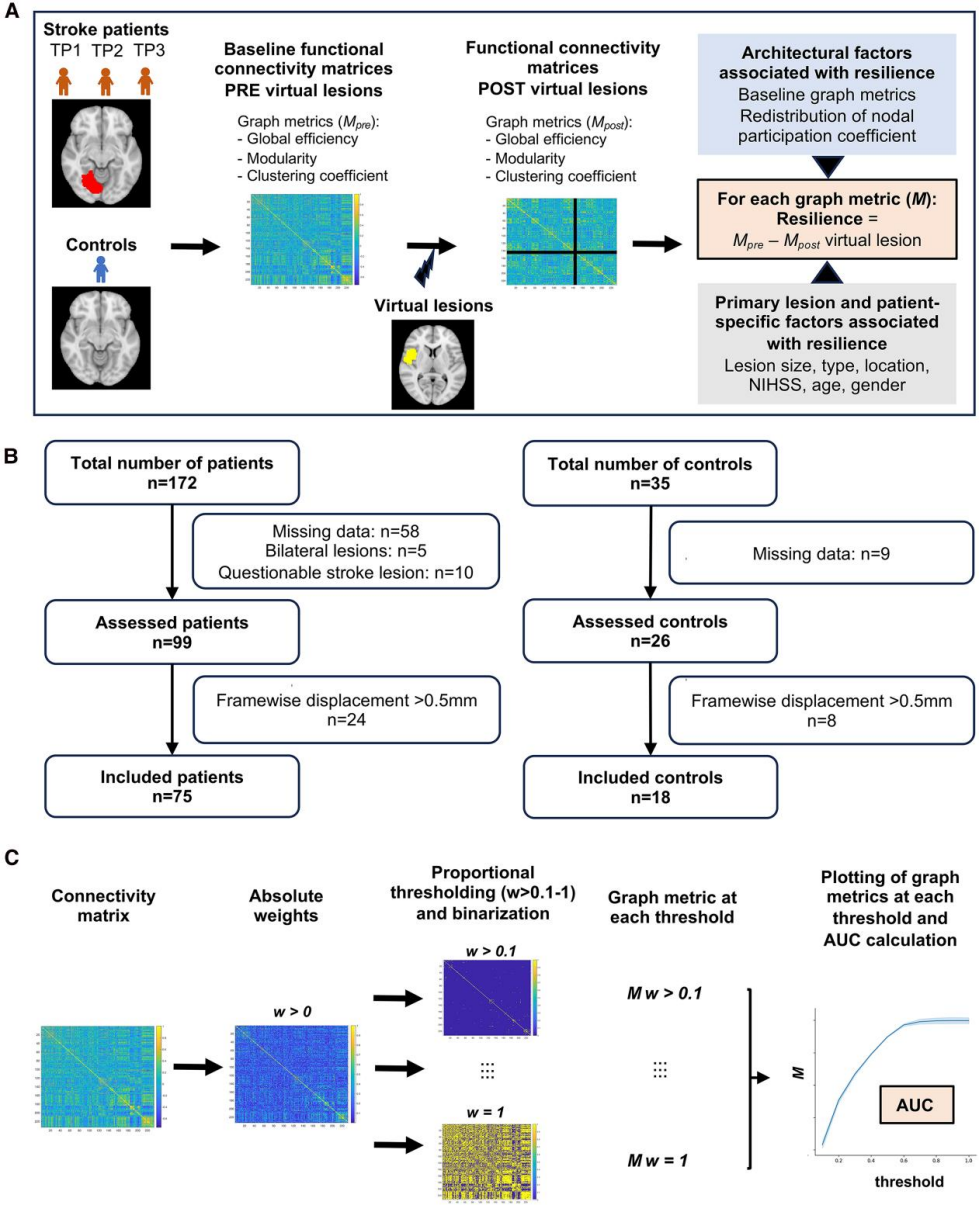
**Experimental design.** (**A**) The primary aim of the study was to determine whether brain network resilience increases following a first stroke compared to controls. Resilience to virtual lesions was assessed by comparing graph properties of connectivity matrices before and after simulating virtual lesions, which involved removing nodes corresponding to clinical stroke masks. In Aims 2 and 3, the association between architectural network factors, primary lesion characteristics and individual patient-specific factors with resilience was examined. (**B**) Patient flow chart: 172 patients and 35 matched controls were screened for inclusion. Data from 75 patients and 18 controls were finally included in the study. (**C**) Pipeline of graph construction: Each connectivity matrix was first thresholded using an absolute threshold *w* > 0 to remove negative weights. Proportional thresholding was then applied from *w* = 0 (no connection) to *w* = 1 (all connections retained), with a density increment of 0.1. Following binarization, graph metrics are calculated at each threshold and plotted to create a curve over increasing densities for each graph metric. The area under the curve was then used for statistical analyses.

The primary goal of this work was to test our hypothesis that stroke-induced reorganization of brain functional architecture enhances resilience to recurrent events (see Fig. 1A for a visual representation of how resilience was defined). Through longitudinal evaluation of a large population of patients, we found, based on the measure of global efficiency and modularity, that resilience is increased during the first year after stroke. This may represent a potential protective measure against recurrent attacks.

Our second aim was to identify architectural characteristics of brain networks that may contribute to resilience, focusing on the distribution on nodal participation coefficient (PC). PC measures the extent to which a node connects to subnetworks outside its own. While global efficiency and modularity are whole-brain network metrics, PC reflects individual node properties. Previous studies suggest that nodes with high PC are network hubs that are highly connected to other subnetworks.^22,30,31^ Here, we hypothesized that, after stroke, PC would decrease in these hub nodes as part of the resilience mechanism. A decrease in PC may help mitigate the risk of significant network disruption if these hubs are affected by a recurrent stroke.

Finally, our third and last aim was to determine patient- and primary stroke-associated characteristics that may be associated with network resilience.

## Materials and methods

### Participants

Acquisition of imaging and clinical data were obtained after all participants had given their written informed consent to participate in accordance with the Declaration of Helsinki and following approval by the Institutional Review Board at Washington University in St. Louis, Missouri. The data collection protocol is described in details in Corbetta *et al*.^32^ and data from this cohort, referred here as ‘Washington cohort’, have been described in previous publications.^21,32-35^ Patients were prospectively recruited at the Barnes-Jewish Hospital in St. Louis from 1 May 2008 to 30 May 2013. A total of 172 patients with first-time ischaemic or haemorrhagic stroke were screened for inclusion, of which 73 were excluded because of missing data (*n* = 58), bilateral lesions (*n* = 5) or questionable stroke lesion (*n* = 10). Remaining 99 patients were assessed for excess head movement during MRI imaging. Twenty-four patients were excluded because more than 60% of volumes had a framewise displacement of >0.5 mm, yielding a total of 75 patients (32 females) for study inclusion.^36,37^ Thirty-five demographically matched healthy control subjects were considered for inclusion. Seventeen controls were excluded because of missing data (*n* = 9) or excess movement (*n* = 8), yielding a total of 18 healthy controls (10 females) (Fig. 1B). Patients’ clinical characteristics are described in Table 1.

**Table 1:** Patients clinical characteristics

	Patients (*N* = 75)	Controls (*N* = 18)
Age	53 (50–59)	55.5 (53–63)
Sex, female (*N*)	32 (43%)	10 (55.5%)
White matter disease (0–9)	1 (0–2)	2 (1–2)
Ischaemic stroke (*N*)	64 (85%)	
Intracerebral haemorrhage (*N*)	11 (15%)	
Lesion side, right (*N*)	39 (52%)	
Lesion size (cm^3^)	9.8 (2.6–42.5)	
NIHSS at hospital	4 (2–9)	
Imaging—days since stroke		
Acute	12.5 (10–15.3)	
3 months	109.5 (100.8–118)	
1 year	382 (373–396)	

### Experimental protocol

For stroke patients, imaging and behavioural data were obtained at up to three time-points (TPs) following index stroke [within 1–2 weeks (TP1), at 3 months (TP2) and at 1 year (TP3)]. Sixty patients had imaging data for all three TPs, whereas 15 patients had imaging data for TP1 and TP2 only. Control subjects were evaluated at two TPs, 3 months apart, to ensure test and re-test reliability of imaging data.

### Imaging and clinical evaluation

Imaging and behavioural evaluations were usually performed on the same day.^21^ Imaging was acquired on a Siemens 3T Tim-Trio scanner at the School of Medicine of the Washington University in St. Louis. Imaging included (i) a structural T_1_-weighted MPRAGE image (repetition time 1950ms, echo time 2.26 ms, flip angle 90°, voxel size 1 × 1 × 1 mm, slice thickness 1 mm); (ii) a T_2_-weighted transverse turbo spin-echo image (repetition time 2500 ms, echo time 435 ms, voxel size 1 × 1 × 1 mm, slice thickness 1 mm); (iii) a FLAIR image (repetition time 7500 ms, echo time 326 ms, voxel size 1.5 × 1.5 × 1.5 mm, slice thickness 1.5 mm); and (iv) Gradient echo planar imaging resting-state functional images (repetition time 2000 ms, echo time 27 ms, 32 contiguous 4 mm slices, 4 × 4 mm in plane resolution), which were acquired while participants were fixing a small cross in a low luminescence environment. Six to eight consecutive resting-state runs of 128 volumes each were acquired for each participant.

Clinical evaluation included the National Institutes of Health Stroke Scale (NIHSS) score. NIHSS is a scale commonly used in clinical practice to assess the functional impact of a stroke. It scores items such as aphasia, dysarthria, motor and visual dysfunction, spatial neglect and ataxia. Higher scores indicate more severe functional deficits, with a maximum score of 42. NIHSS has been used to assess behaviour in previous studies investigating brain network changes in the acute stroke phase.^23^ When the NIHSS score was not available for TP1, NIHSS determined at admission was considered.

### Lesion analysis

**Figure 2:**
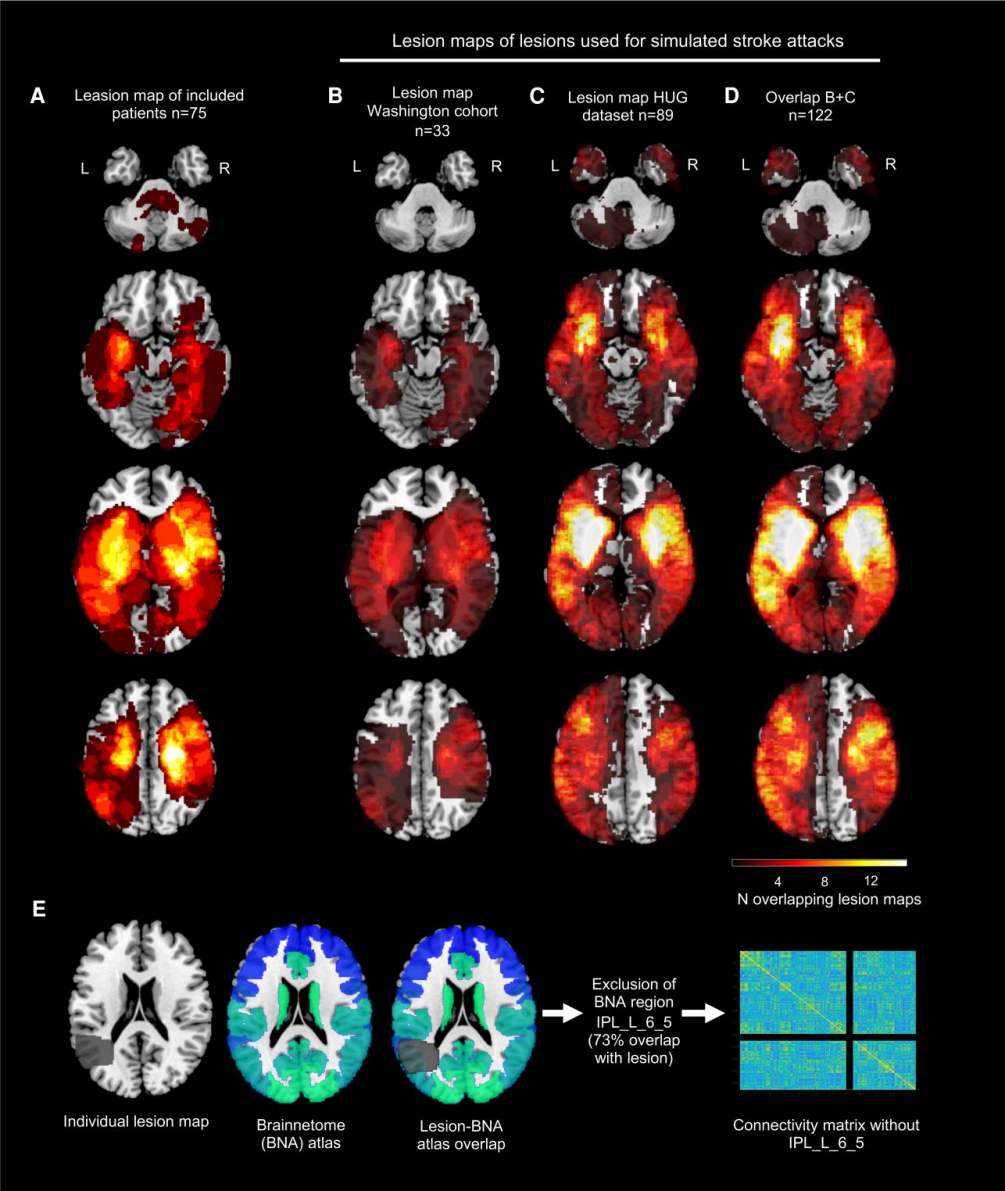
**Lesion maps and lesion removal from connectivity matrices.** (**A**) Primary lesion map of all included patients (*N* = 75). (**B**) Lesion map of lesions used from the Washington cohort (*N* = 33) to simulate virtual strokes. (**C**) Lesion map of lesions used from the in-house dataset (*N* = 89) to simulate virtual strokes. (**D**) Overlap of **B** + **C** corresponding to all lesion masks used to simulate virtual strokes (*N* = 122). (**E**) Removal of Brainnetome regions with >50% overlap with index stroke lesions: individual lesion maps were overlapped with corresponding atlased connectomes. Brainnetome atlas regions with more than 50% overlap with the lesion mask were removed from the connectivity matrix.

Lesions were manually segmented on structural MRI images (T_1_, T_2_, FLAIR) obtained between 1 and 3 weeks post stroke, using the Analyze biomedical imaging software system.^21,32,38^ Segmented lesions were reviewed by two board-certified neurologists and were classified automatically based on their overlap with three masks: grey matter, white matter and subcortical regions including basal ganglia and thalamus. A lesion heat map of all included patients shows the distribution of primary stroke lesions across vascular territories (Fig. 2A).

### Imaging data preprocessing

Imaging data were preprocessed using SPM12 and in-house MATLAB scripts.^29,39^ The first five volumes were discarded to allow magnetization equilibrium. Remaining resting-state functional images were then realigned to the first image. Anatomical T_1_ images were spatially co-registered to the mean functional image obtained after spatial realignment. T_1_ images were then segmented into grey matter, white matter and cerebrospinal fluid maps. The grey matter map of each subject was atlased in native space, into 246 regions, according to the Brainnetome atlas.^40^ For this step, the Brainnetome atlas was reverse normalized to the T_1_ grey matter mask of each subject. The resulting maps were co-registered to corresponding functional data to obtain atlased functional images in subjects’ native space. All atlased T1 and functional images were visually inspected. When atlasing of functional images was suboptimal using the T_1_ image from the same TP, a T_1_ image from a different TP was used for co-registration and atlasing.

### Extraction of brain signals and connectivity matrices construction

Time courses were linearly detrended at each voxel and averaged for each atlas region.^29^ The six motion parameters, their first derivatives and the average cerebrospinal fluid signal were regressed out. To further decrease noise and outlying spikes, time courses were winsorized to the 5th and 95th percentiles and filtered into four frequency sub-bands using a wavelet transform. The frequency range 0.03 < *f* < 0.06 Hz was considered for further analyses. Connectivity matrices were obtained by computing Pearson correlation coefficients between the 246 Brainnetome atlas regions. Regions with signal drop-out (corresponding to a maximum of 16 small regions in artefact prone inferior temporal regions and deep grey matter) and for each patient, Brainnetome regions with >50% overlap with their stroke lesion were removed from the connectivity matrix^21^ (Fig. 2E).

### Graph metrics

Resilience of brain networks may be determined using whole-brain graph metrics.^25,29^ As mathematically described in Supplementary material, following graph metrics (*M*) were computed to assess resilience to recurrent events and network properties that may support resilience: network global efficiency (*E*_glob_), modularity, clustering coefficient (CC) and nodal PC. These metrics were chosen as surrogates of intrinsic brain network organization.^13,14^ Global efficiency is a measure of functional integration that provides a measure of information transfer across all nodes of the network (here, Brainnetome regions).^41^ Modularity quantifies the degree to which a network is divided into distinct modules. Networks with high modularity have a greater number of within-module connections and fewer between-module connections. We used the 17-network parcellation described by Yeo *et al*.^42^ as an *a priori* definition of modules, with nodes within those modules defined by Brainnetome regions. Whole-brain average CC was assessed as another measure of network segregation, reflecting how well nodes are grouped within the graph.^43^ We finally used nodal PC to identify brain hubs. At the nodal level, PC measures how evenly a node's connections are distributed across modules.^44^ Nodes with higher PCs have more inter-module edges and can be considered hubs within their respective modules.^30,45^

### Graph analysis

Graphs were constructed as described in Fig. 1C. First, each connectivity matrix was thresholded with an absolute threshold *w* > 0 to remove negative weights. The calculation of graph metrics was performed on binarized matrices, which implied thresholding weighted connectivity matrices. To avoid the use of an arbitrary threshold and examine graph properties over a range of edge densities, a proportional thresholding *w* was applied. This process involved successively thresholding a weighted connectivity matrix by incrementally increasing the threshold by 0.1 starting from *w* = 0 (no connection) to *w* = 1 (all connections retained).^29^ At the end of this processing pipeline, each weighted connectivity matrix was represented by 10 binarized connectivity matrices (one for each density range). Graph metrics were then computed for each density range, resulting in 10 values that were plotted to create a curve over increasing densities (see Supplementary material for a mathematical description of graph measures). We then derived the area under the curve over the entire connectivity density range (δ; [0–1]), which was used as a proxy of each graph metric *M* and to perform statistical analyses, as proposed in van Assche *et al*.^29^


$$\mathrm{AUC}(M)=\int_{0}^{1}M(\delta )\;$$

Before stroke simulations, we evaluated graph metrics for controls at TP1 and TP2. Since there was no statistical difference between the control TPs, only control TP1 values were used for further analyses.

### Attack simulation and resilience definition

We defined resilience as the ability of brain networks to maintain their core organization including balanced functional integration and specialization, following an acute lesion. Given the unpredictable nature of strokes in the clinical setting, we simulated recurrent strokes using virtual lesions. These lesions were generated by removing Brainnetome regions of interest (ROI) from whole-brain connectivity matrices, if they overlapped with patient-derived stroke masks. An ROI was considered lesioned if at least 50% of its voxels overlapped with the lesion mask. To capture the diversity of potential recurrent strokes, lesion masks were sourced from two stroke cohorts. First, we analysed the 75 patients from the Washington cohort included in our study, of which 33 masks overlapped with at least 50% of one Brainnetome ROI, covering 52% of Brainnetome regions (Fig. 2B). We further considered lesions from 144 in-house consecutive stroke patients,^46^ 89 of which overlapped with at least 50% of one Brainnetome ROI, covering 78% of Brainnetome regions (Fig. 2C). Combined, these datasets included 122 lesion masks, covering 82% of the Brainnetome atlas (Fig. 2D). The median number of Brainnetome regions included in each lesion mask was six (interquartile range 2–11). Up to 122 virtual attacks were used to test and compare resilience in patients (at all three TPs) and control subjects. Some patients were subjected to less than 122 attacks, since one or more virtual lesion fell within their primary stroke (122 attacks: *n* = 56, 121 attacks: *n* = 4, 120 attacks: *n* = 2, 119 attacks: *n* = 2, 118 attacks: *n* = 1, 115 attacks: *n* = 2, 114 attacks: *n* = 1, 113 attacks: *n* = 2, 112 attacks: *n* = 1, 110 attacks: *n* = 2, 108 attacks: *n* = 1, 97 attacks: *n* = 1).

Computationally, resilience was defined for every graph metric *M*, as the difference between pre-attack (baseline) and post-attack values:


$$R(M)=\;M_{\mathrm{pre}}-\;M_{\mathrm{post}}$$

*R* values of patients and controls were further normalized to the average control *R* value.

This normalization provides a fractional deviation from the expected resilience observed in the control population, enabling a more accurate estimation of the effect size:


$$R_{\mathrm{norm}}(M)=\;1-\frac{(M_{\mathrm{observed}\;\mathrm{pre}}-M_{\mathrm{observed}\;\mathrm{post}})\;}{\mathrm{mean}(M_{\mathrm{control}\;\mathrm{pre}}-\;M_{\mathrm{control}\;\mathrm{post}})}$$

where *M*_observed_ is the measured metric in each individual, pre- and post-attack and mean *M*_control_ is the mean pre–post-attack metric in the control population.

*R*_norm_(*E*_glob_), *R*_norm_(modularity) and *R*_norm_(CC) were used as resilience metrics for the corresponding graph measures.

### Statistical analysis

Statistical analyses were performed using Python (3.11.9), R (4.1.0) and SPSS Statistics Version 27.

#### Network resilience dynamics during stroke recovery

The primary aim of the study was to determine whether a first stroke induces changes in whole-brain networks that enhance resilience to future lesions.

Patient and control groups were compared using mixed-effects models with *R*_norm_(*E*_glob_), *R*_norm_(modularity) or *R*_norm_(CC), as dependent variables. Group [control versus patient (P)] and TP within group (control TP1 versus P TP1 versus P TP2 versus P TP3) were chosen as fixed effects and subject as random effect. To correct for multiple comparisons, pairwise comparisons of TPs were adjusted using the Benjamini–Hochberg false discovery rate (FDR) correction.

Following the main analysis, we sought to exclude potential statistical bias arising from unequal group sizes, as the patient group (*n* = 75) was larger than the control group (*n* =18). To address this, we randomly selected 18 patients and compared them to the 18 controls. This comparison was repeated for four different sets of 18 random patients, using a mixed-effects model as described above.

### Architectural specificities of stroke patient networks that may sustain resilience

The second aim of the study was to determine architectural characteristics of brain networks, that may support resilience.

#### Global efficiency, modularity and CC

We first assessed, whether baseline graph measures before stroke lesion simulation differed between patients and controls. Both groups were compared using mixed-effects models with baseline global efficiency, modularity and CC as dependent variables. Group and TP within group (control TP1 versus P TP1 versus P TP2 versus P TP3) were chosen as fixed effects and subject as random effect. To correct for multiple comparisons, pairwise comparisons of TPs were adjusted using the Benjamini–Hochberg FDR correction.

#### Nodal PC

We further examined the distribution of nodal PC, as a potential mechanism underlying increased network resilience.

First, we assessed the average nodal PC across the entire network for both patients and controls. A mixed-effects model was used with average network PC as dependent variable, group and TP within group as fixed effects and subject as random effect.

Next, we identified the 10 nodes with highest PC values in controls, referred to as hubs.^45^ PC values for these nodes were compared between patients and controls, using a mixed-effects model with nodal PC as dependent variable, group, node and TP within group as fixed effects and subject as random effect.

We then identified the 10 nodes with lowest PC values in controls and compared these values between patients and controls using the same mixed-effects model.

Finally, we correlated patients’ PC for the 10 highest PC nodes (hubs), across all TPs with *R*_norm_(*E*_glob_) or *R*_norm_(modularity) using Spearman’s rank correlation. Similarly, we calculated Spearman correlations between *R*_norm_(*E*_glob_) or *R*_norm_(modularity) and patients’ PC for the 10 nodes with the lowest PC values.

Correction for multiple comparisons was applied using the Benjamini–Hochberg FDR correction.

#### Patient and primary lesion factors associated with network resilience

The last aim of the study was to evaluate patient and primary lesion factors that are associated with network resilience. Two separate mixed-effects models were created with either *R*_norm_(modularity) or *R*_norm_(*E*_glob_) as dependent variables and subject as random effect. TP, age, gender and NIHSS score were used as patient-specific fixed effects. Lesion size, type (ischaemic or haemorrhagic) and location (subcortical, cortical, cortico-subcortical, white matter, brainstem or cerebellum) were used as lesion-specific fixed factors. Correction for multiple comparisons was applied using the Benjamini–Hochberg FDR correction.

*R*_norm_(*E*_glob_) and *R*_norm_(modularity) were further averaged across all TPs for each patient and stroke location. Resulting values were compared between locations using a one-way ANOVA with Bonferroni correction.

## Results

Seventy-five patients with a first-ever ischaemic or haemorrhagic stroke were evaluated and compared to 18 demographically matched control subjects. Resting-state functional MRI was acquired at three TPs: TP1 (within 2 weeks of the stroke), TP2 (at 3 months post stroke) and TP3 (at 1 year post stroke).

### Network resilience dynamics during stroke recovery

**Figure 3:**
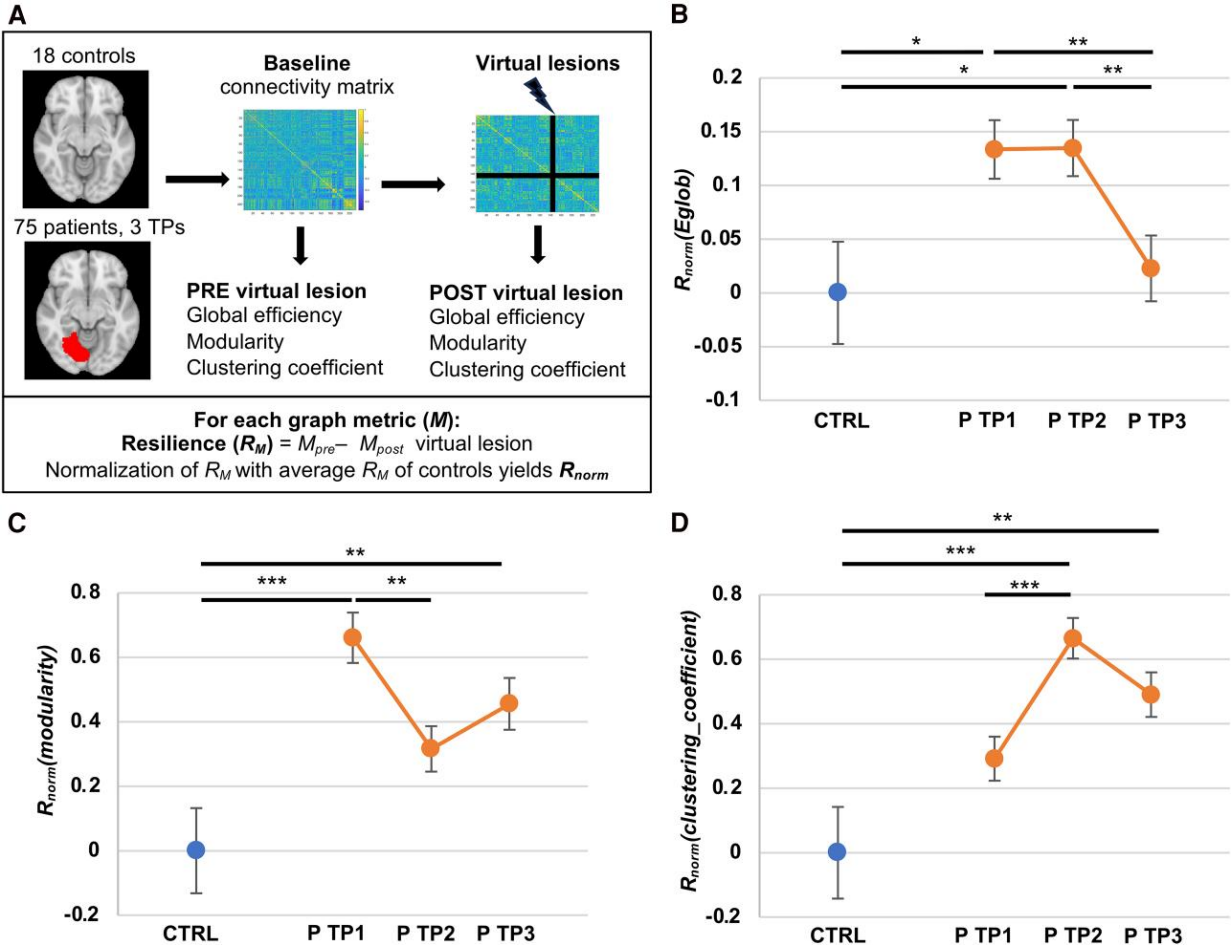
**Evolution of resilience to virtual lesions over time.** (**A**) Resilience to virtual lesions was assessed in 18 control subjects and 75 stroke patients at three TP following stroke onset: TP1 (within 2 weeks of stroke onset), TP2 (3 months post stroke) and TP3 (1 year post stroke). Whole-brain graph metrics calculated both before and after virtual lesions were applied to the functional connectivity matrices. Resilience was defined as the difference between the post-lesion and pre-lesion graph metrics. (**B**) Resilience of global efficiency [*R*_norm_(*Eglob*)] following up to 122 virtual lesions in control subjects (CTRL, *N* = 18) and patients (P, *N* = 75) at all three TPs [group effect: *F*(1,27367) = 2.97, *P* = 0.085; TP (group) effect: *F*(2,27367) = 4.99, *P* = 0.007]. (**C**) Resilience of modularity [*R*_norm_(modularity)] following up to 122 virtual lesions in control subjects (*N* = 18) and patients (*N* = 75) at all three TPs [group effect: *F*(1,27367) = 9.59, *P* = 0.002; TP (group) effect: *F*(2,27367) = 5.63, *P* = 0.004]. (**D**) Resilience of mean CC [*R*_norm_(clustering_coefficient)] following up to 122 virtual lesions in control subjects (*N* = 18) and patients (*N* = 75) at all three TPs [group effect: *F*(1,27367) = 12.35, *P* < 0.001; TP (group) effect: *F*(2,27367) = 8.25, *P* < 0.001]. Significance was evaluated using mixed-effects models [with group (CTRL versus P) and TP within group (TP1 CTRL, TP1 P, TP2 P, TP3 P) as fixed factors]. Benjamini–Hochberg FDR correction was applied for pairwise comparisons: * *P* < 0.05, ** *P* < 0.01, *** *P* < 0.001. Error bars correspond to the standard error of the mean.

The first aim of the study was to examine brain network resilience to recurrent strokes. We calculated changes in graph metrics representing core brain network organization following simulation of up to 122 virtual lesions in first-stroke patients and matched controls. Resilience was assessed at up to three TPs during recovery of a first stroke, with the normalized difference between pre- and post-virtual lesion graph metrics serving as surrogate of resilience (Fig. 3A).

Resilience was generally higher in stroke patients compared to controls, with recurrent virtual lesions having a smaller impact on network global efficiency and modularity in patients, though variations were observed across TPs. For global efficiency, we observed a significant TP within group effect [group effect: *F*(1,27367) = 2.97, *P* = 0.085; TP (group) effect: *F*(2,27367) = 4.99, *P* = 0.007]. Compared to controls (0.000 ± 0.048), *R*_norm_(*E*_glob_) was significantly higher in patients at TP1 (0.133 ± 0.027, *P* = 0.04 FDR corrected) and TP2 (*R*_norm_(*E*_glob_) 0.135 ± 0.026, *P* = 0.04 FDR corrected) but not TP3 [*R*_norm_(*E*_glob_) 0.023 ± 0.031, *P* = 0.854 FDR corrected] (Fig. 3B). No significant difference in resilience was observed between patients’ TP1 and TP2 (*P* = 0.972 FDR corrected). However, *R*_norm_(*E*_glob_) was significantly higher at TP1 compared to TP3 (*P* = 0.009 FDR corrected) and at TP2 compared to TP3 (*P* = 0.009 FDR corrected) (Fig. 3B).

We observed a similar TP (group) effect for resilience of modularity [group effect: *F*(1,27367) = 9.59, *P* = 0.002; TP (group) effect: *F*(2,27367) = 5.63, *P* = 0.004]. *R*_norm_(modularity) was higher after virtual attacks in patients at TP1 (0.661 ± 0.078, *P* < 0.001 FDR corrected) and TP3 (0.456 ± 0.080, *P* = 0.007 FDR corrected), compared to controls (0.000 ± 0.132) (Fig. 3C). Despite a positive trend at TP2, *R*_norm_(modularity) was not significantly different from controls at this TP [*R*_norm_(modularity) 0.316 ± 0.071, *P* = 0.073 FDR corrected]. *R*_norm_(modularity) was not significantly different between patients’ TP1 and TP3 (*P* = 0.073 FDR corrected) nor between TP2 and TP3 (*P* = 0.202 FDR corrected). However, it was higher at TP1 compared to TP2 (*P* = 0.003 FDR corrected) (Fig. 3C).

Notably, more patients (*n* = 75) were included in the comparison than controls (*n* = 18). To account for potential bias from unequal group sizes, we randomly selected 4 sets of 18 patients for comparison with the 18 controls. Mixed linear models confirmed a significant (*P* < 0.05) group effect, with patients showing higher resilience than controls for both global efficiency and modularity, suggesting that the unequal group sizes did not bias the main analysis.

As a complementary measure, we evaluated resilience in terms of CC. As for modularity, increased resilience was observed in patients compared to controls [group effect: *F*(1,27367) = 12.35, *P* < 0.001; TP (group) effect: *F*(2,27367) = 8.25, *P* < 0.001]. *R*_norm_(CC) was significantly lower in controls (0.000 ± 0.142) compared to patients at TP2 (0.665 ± 0.063, *P* < 0.001 FDR corrected) and TP3 (0.490 ± 0.069, *P* = 0.002), with a non-significant trend at TP1 (0.291 ± 0.068, *P* = 0.056 FDR corrected) (Fig. 3D). Additionally, a significant difference was observed between patients’ TP1 and TP2 (*P* < 0.001 FDR corrected).

### Architectural specificities of stroke patient networks that may sustain resilience

We further considered intrinsic architectural specificities that may underlie the higher resilience of patient brain networks to virtual strokes.

#### Global efficiency, modularity and CC

**Figure 4:**
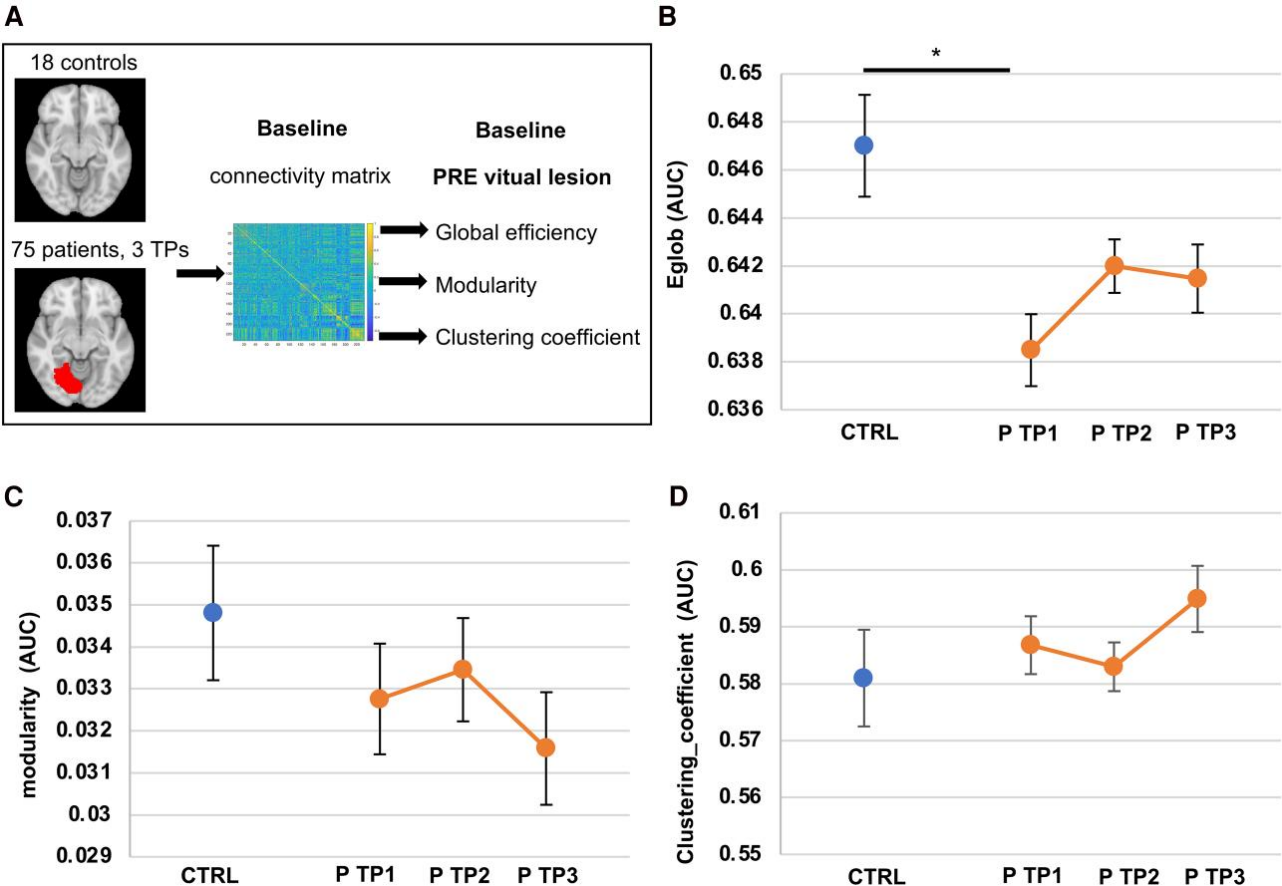
**Baseline network characteristics over time.** (**A**) Baseline whole-brain graph metrics were compared between controls (*N* = 18) and first-stroke patients (*N* = 75) prior to recurrent stroke simulation. (**B**) Baseline whole-brain global efficiency (*Eglob*) of control subjects (CTRL, *N* = 18) and patients (P, *N* = 75) at TP1 (within 2 weeks of stroke onset), TP2 (at 3 months of stroke onset), and TP3 (at 1 year of stroke onset) [group effect: *F*(1,224) = 5.48, *P* = 0.021; TP (group) effect: *F*(2,224) = 2.12, *P* = 0.124]. (**C**) Baseline whole-brain modularity in control subjects (*N* = 18) and patients (*N* = 75) at all three TPs [group effect: *F*(1,224) = 0.72, *P* = 0.397; TP (group) effect: *F*(2,224) = 0.53, *P* = 0.592]. (**D**) Baseline mean CC in control subjects (*N* = 18) and patients (*N* = 75) at all three TPs [group effect: *F*(1,224) = 0.50, *P* = 0.480; TP (group) effect: *F*(2,224) = 1.40, *P* = 0.249]. Significance was evaluated using mixed-effects models [with group (CTRL versus P) and TP within group (TP1 CTRL, TP1 P, TP2 P, TP3 P) as fixed factors]. Benjamini–Hochberg FDR correction was applied for pairwise comparisons: * *P* < 0.05. Error bars correspond to the standard error of the mean.

We first evaluated whether baseline graph measures, prior to stroke lesion simulation, were associated with post-attack metrics. To this end, we assessed baseline global efficiency, modularity and CC in the whole-brain connectivity matrices of patients and controls at each time point before virtual lesions (Fig. 4A).

Regarding global efficiency, we observed a significant group effect [group effect: *F*(1,224) = 5.48, *P* = 0.021; TP (group) effect: *F*(2,224) = 2.12, *P* = 0.124]. Indeed, at TP1, patients displayed significantly lower global efficiency compared to controls (0.638 ± 0.001 versus 0.647 ± 0.002, *P* = 0.012 FDR corrected) (Fig. 4B). Global efficiency was not significantly different between patients and controls at TP2 (0.642 ± 0.001 versus 0.647 ± 0.002, *P* = 0.088 FDR corrected) and TP3 (0.641 ± 0.001 versus 0.647 ± 0.002, *P* = 0.088 FDR corrected). There was no significant difference in global efficiency across TPs in patients. To rule out the possibility that lower baseline global efficiency at TP1 drives the increased resilience observed in patients at this TP, we calculated the correlation between baseline global efficiency at TP1 and resilience [*R*_norm_(*E*_glob_)]. No significant correlation was found (correlation coefficient −0.103 *P* = 0.378).

Following a first stroke, modularity did not differ significantly between controls and patients at any TP nor across patients’ TPs [patients TP1 0.033 ± 0.001, TP2 0.033 ± 0.001, TP3 0.032 ± 0.001, controls 0.035 ± 0.002, group effect: *F*(1,224) = 0.72, *P* = 0.397; TP (group) effect: *F*(2,224) = 0.53, *P* = 0.592] (Fig. 4C).

Similarly, there was no significant difference in CC between controls and patients, nor across patients’ TPs [TP1 0.587 ± 0.005, TP2 0.583 ± 0.004, TP3 0.595 ± 0.006, controls 0.581 ± 0.008, group effect: *F*(1,224) = 0.50, *P* = 0.480; TP (group) effect: *F*(2,224) = 1.40, *P* = 0.249] (Fig. 4D).

#### Nodal PC

We then examined the distribution of nodal PC, as a potential mechanism for increased network resilience.

PC measures how much a node connects to other modules outside its own.^22,30,44^ Nodes with high PC are considered hubs, as they are well-connected to other subnetworks. We hypothesized that patients may have lower PC values in these hubs compared to controls to limit the network-wide impact of recurrent strokes affecting these nodes. We also expected a compensatory increase in PC in non-hub nodes to maintain whole-brain connectivity.

**Figure 5:**
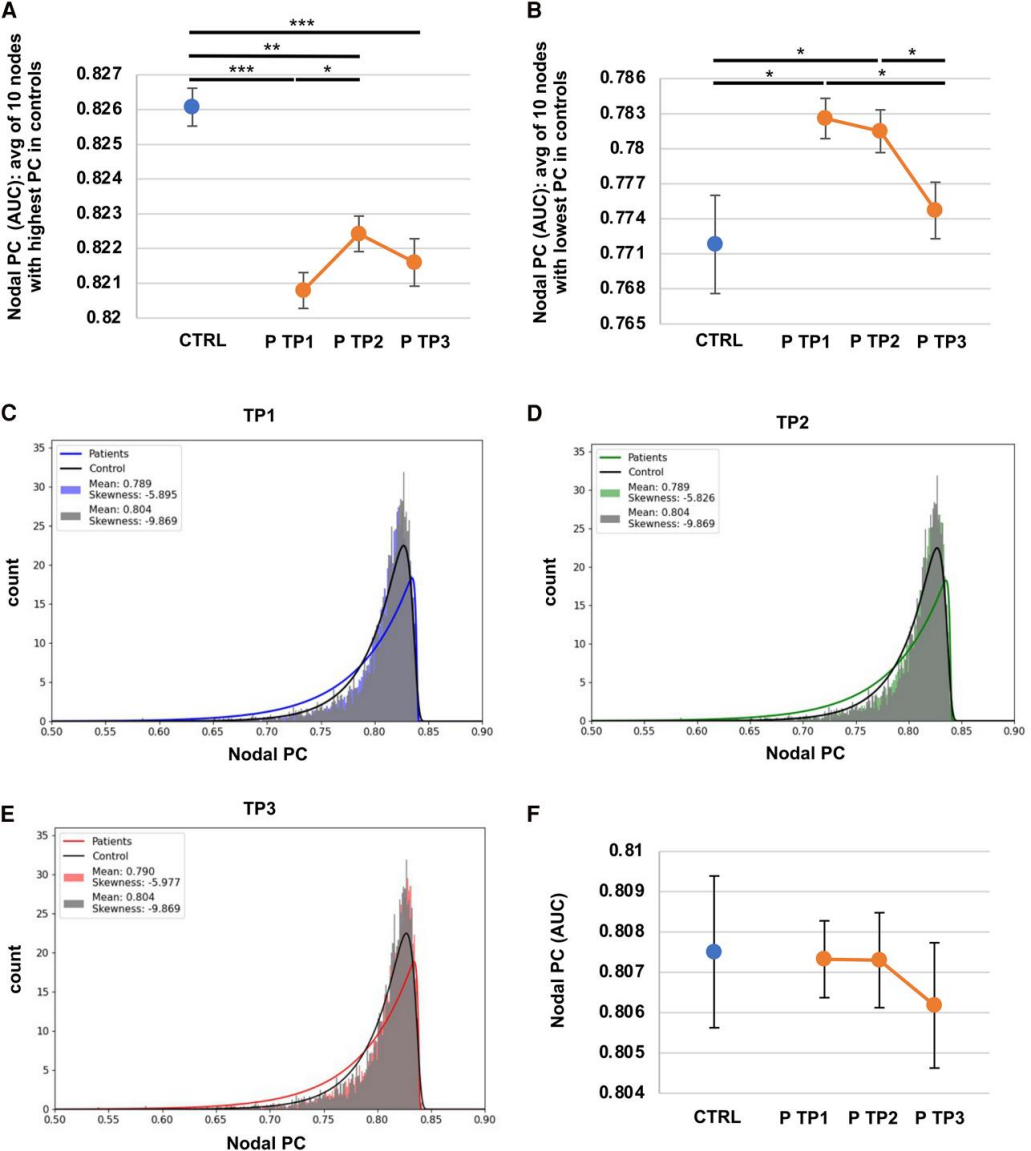
**Redistribution of nodal PC after a first stroke.** (**A**) Average (avg) nodal PC of the 10 nodes with the highest PC values in control subjects (hubs) for controls (CTRL, N = 18) and patients (P, *N* = 75) at TP1 (within 2 weeks of stroke onset), TP2 (at 3 months of stroke onset), and TP3 (at 1 year of stroke onset) [group effect: *F*(1,2253) = 16.19, *P* < 0.001; TP (group) effect: *F*(2,2253) = 2.44, *P* = 0.088]. (**B**) Average nodal PC of the 10 nodes with the lowest PC values in control subjects in controls (*N* = 18) and patients (*N* = 75) at all three TPs [group effect: *F*(1,2232) = 3.79, *P* = 0.053; TP (group) effect: *F*(2,2232) = 4.28, *P* = 0.014]. (**C**) Nodal PC distribution in control subjects (*N* = 18) and patients (*N* = 75) with corresponding fitted exponentiated Weibull distributions at TP1 and TP2 (**D**) and TP3 (**E**). (**F**) Whole-brain mean nodal PC in control subjects (*N* = 18) and patients (*N* = 75) at all three TPs [group effect: *F*(1,224) = 0.05, *P* = 0.816; TP (group) effect: *F*(1,224) = 0.28, *P* = 0.759]. Significance was evaluated using mixed-effects models [with group (CTRL versus P) and TP within group (TP1 CTRL, TP1 P, TP2 P, TP3 P) as fixed factors]. Benjamini–Hochberg FDR correction was applied for pairwise comparisons: * *P* < 0.05, ** *P* < 0.01, *** *P* < 0.001. Error bars correspond to the standard error of the mean.

We first assessed the PC values of the 10 Brainnetome atlas nodes with the highest PC in controls, referred to as brain hubs^45^ (Supplementary Table 1). PC values of these hubs were significantly lower in patients than in controls [group effect: *F*(1,2253) = 16.19, *P* < 0.001; TP (group) effect: *F*(2,2253) = 2.44, *P* = 0.088] (controls 0.826 ± 0.001 versus patients at TP1 0.821 ± 0.001, *P* < 0.001; TP2 0.822 ± 0.001, *P* = 0.004; TP3 0.822 ± 0.001, *P* < 0.001 FDR corrected) (Fig. 5A). In patients, PC of these 10 nodes was lower at TP1 versus TP2 (*P* = 0.04 FDR corrected). Importantly, a significant negative correlation was found between PC of these 10 nodes and (i) *R*_norm_(*E*_glob_) (Spearman’s rho correlation coefficient −0.079, *P* < 0.001) and (ii) *R*_norm_(modularity) (Spearman’s rho correlation coefficient −0.084, *P* < 0.001) in patients. This indicates that lower PC values for these 10 nodes were associated with higher resilience.

Conversely, the 10 nodes with the lowest PC in controls (Supplementary Table 2) showed overall higher values in patients [group effect: *F*(1,2232) = 3.79, *P* = 0.053; TP (group) effect: *F*(2,2232) = 4.28, *P* = 0.014], with significance observed at TP1 and TP2, but not TP3 (controls 0.772 ± 0.004; patients at TP1 0.783 ± 0.002, *P* = 0.036; TP2 0.781 ± 0.050, *P* = 0.036; TP3 0.774 ± 0.002, *P* = 0.601 FDR corrected) (Fig. 5B). In patients, PC of these 10 nodes were significantly lower at TP3 compared to TP1 (*P* = 0.036) and TP2 (*P* = 0.036). Moreover, there was a significant positive correlation between PC of these 10 nodes in patients and (i) *R*_norm_(*E*_glob_) (correlation coefficient 0.089, *P* < 0.001) and (ii) *R*_norm_(modularity) (correlation coefficient 0.335, *P* < 0.001). Therefore, higher PC values for these 10 nodes were associated with higher resilience.

We then fitted an exponentiated Weibull distribution to the nodal PC distribution of controls and patients (Fig. 5C–E). This analysis confirmed that nodes with lower PC in controls exhibited higher PC values in patients up to an intersection point, after which patients showed lower PC values than controls. Additionally, the redistribution of nodal PC was reflected in more negative skewness of the fitted curve in controls than in patients. This redistribution did not affect the mean PC values, which were calculated by averaging PC values of all network nodes. Indeed, there was no significant difference in mean PC values between controls and patients [controls mean PC 0.808 ± 0.002; patients mean PC at TP1 0.807 ± 0.001, TP2 0.807 ± 0.010, TP3 0.806 ± 0.01; group effect: *F*(1,224) = 0.05, *P* = 0.816; TP (group) effect: *F*(1,224) = 0.28, *P* = 0.759] (Fig. 5F).

### Patient and primary lesion factors associated with resilience

Finally, we determined patient and primary lesion characteristics associated with resilience to strokes. For this purpose, a mixed linear model was built with both patient- and lesion-specific variables as fixed effects and resilience [*R*_norm_(*E*_glob_) or *R*_norm_(modularity)] as dependent variable.

#### Patient-specific characteristics associated with resilience

Patient characteristics tested in the analysis included age at the time of stroke, gender and NIHSS scoring. NIHSS is widely used in clinical practice to assess the functional impact of a stroke. Higher scores imply greater functional deficits. The cohort mean NIHSS at TP1 was six (SD = 6). NIHSS was positively associated with brain network resilience after stroke in terms of global efficiency [estimate of fixed effect on *R*_norm_(*E*_glob_): 0.0101, 95% confidence interval (CI) 0.0026–0.0176, *P* = 0.014 FDR corrected], but not modularity [estimate of fixed effect on *R*_norm_(modularity): 0.0040, 95% CI −0.0166–0.0245, *P* = 0.823] (Table 2). This suggests that patients with more severe clinical deficits following the primary stroke tended to maintain higher global efficiency after virtual recurrent strokes.

**Table 2:** Patient and primary lesion factors associated with resilience

	*R*_norm_(*Eglob*) Estimate of fixed effect	Std. Error	95% confidence interval	*P*-value (FDR corrected)	*R*_norm_(modularity) Estimate of fixed effect	Std. Error	95% confidence interval	*P*-value (FDR corrected)
NIHSS	0.0101	0.0038	0.0026–0.0176	0.014	0.0040	0.0105	−0.0166–0.0245	0.823
Age	−0.0137	0.0019	−0.0175-−0.0099	<0.001	0.0334	0.0053	0.0223–0.0438	<0.001
Gender				0.050				<0.001
Male	−0.0729	0.0352	−0.1421-−0.0038		0.4339	0.0964	0.2449–0.6228	
Female	0	0			0	0		
Lesion size	0.46^E-5^	0.4^E-5^	−0.4^E-5^–1.3^E-5^	0.292	0.14^E-5^	1.19^E-5^	−2.19^E-5^–2.48^E-5^	0.904
Lesion type								
Ischaemic	−0.2010	0.0535	−0.3059-−0.0961	<0.001	0.2947	0.1462	0.0081–0.5814	0.062
Haemorrhagic	0	0			0	0		
Primary lesion site				<0.001				<0.001
Subcortical	0.0545	0.0743	−0.0912–0.2001		1.7586	0.2028	1.3611–2.1562	
Cortical	0.3205	0.0663	0.1906–0.4504		1.2302	0.1810	0.8754–1.5850	
Cortico-subcortical	0.2373	0.0885	0.0639–0.4107		2.7513	0.2417	2.2775–3.2250	
White matter	0.3609	0.0895	0.1855–0.5364		1.8281	0.2446	1.3487–2.3074	
Brainstem	0.1282	0.0814	−0.0314–0.2877		0.9762	0.2223	0.5404–1.4120	
Cerebellum	0	0			0	0		

Mean age at stroke onset was 53 years old (SD = 9 years old). Age was negatively associated with resilience to virtual attacks when considering global efficiency (estimate of fixed effect −0.0137, 95% CI −0.0175 to −0.0099, *P* < 0.001). Conversely, it was positively associated with resilience in terms of modularity (estimate of fixed effect 0.0334, 95% CI 0.0223–0.0438 *P* < 0.001; Table 2).

We observed a positive association between female sex and resilience of global efficiency [estimate of fixed effect on *R*_norm_(*E*_glob_) in males: −0.0729, 95% CI −0.1421 to −0.0038, *P* = 0.050 FDR corrected], whereas there was a positive association between resilience for modularity and male sex [estimate of fixed effect on *R*_norm_(modularity) in males: 0.4339, 95% CI 0.2449–0.6228, *P* < 0.001 FDR corrected] (Table 2).

#### Lesion-specific characteristics associated with resilience

Lesion-specific characteristics included index stroke size, type (ischaemic or haemorrhagic) and location.

Primary lesion size had a mean volume of 29 cm^3^ (SD = 47 cm3). Size was not significantly associated with network resilience following virtual attacks [estimate of fixed effect on *R*_norm_(*E*_glob_): 0.46 × 10^−5^, 95% CI −0.4 to 1.3 × 10^−5^, *P* = 0.292; estimate of fixed effect on *R*_norm_(modularity): 0.14 × 10^−5^, 95% CI −2.19 to 2.48 × 10^−5^, *P* = 0.904] (Table 2).

Primary lesions included 11 haemorrhagic (15%) and 64 ischaemic (85%) strokes. Lesion type was associated with resilience. The association between resilience of global efficiency and stroke type was stronger for haemorrhagic than ischaemic strokes [estimate of fixed effect on *R*_norm_(*E*_glob_) for ischaemic strokes: −0.2010, 95% CI −0.3059 to −0.0961, *P* < 0.001 FDR corrected]. Although there was a trend suggesting a stronger association between resilience of modularity and ischaemic stroke, this did not reach statistical significance [estimate of fixed effect on *R*_norm_(modularity) for ischaemic strokes: 0.2947, 95% CI 0.0081–0.5814, *P* = 0.062 FDR corrected] (Table 2).

**Figure 6:**
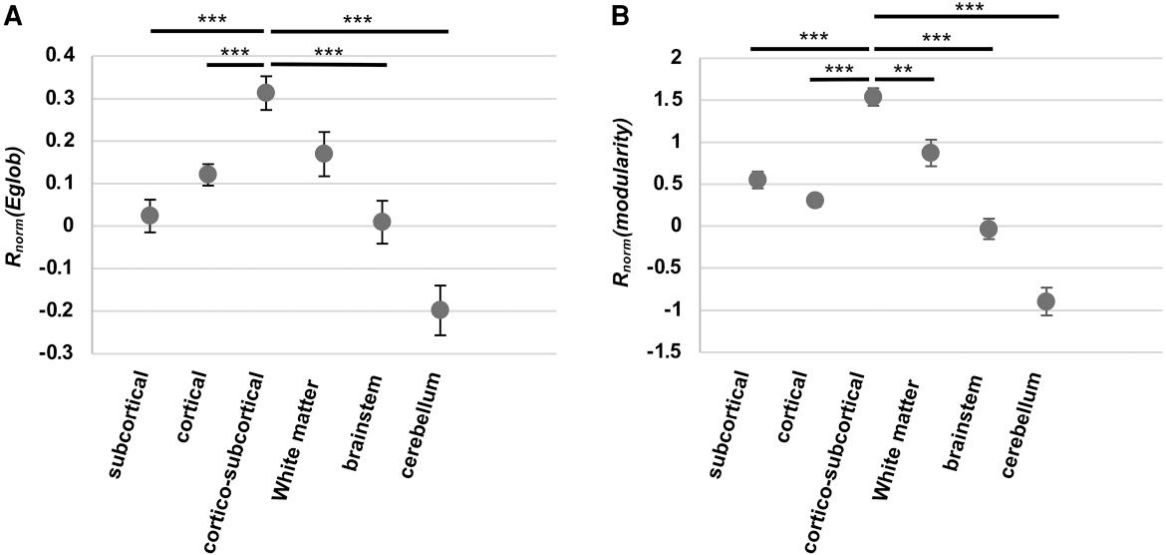
**Resilience according to primary stroke location.** (**A**) Resilience of global efficiency [*R*_norm_(*Eglob*)] according to primary stroke location. (**B**) Resilience of modularity [*R*_norm_(modularity)] according to primary stroke location. Mean resilience pooled for all TPs (at 2 weeks, 3 months, and 1 year of stroke onset) was compared between primary stroke locations using a one-way ANOVA with Bonferroni correction for multiple comparisons [total number of attacks: subcortical strokes = 4704; cortical strokes = 9187; cortico-subcortical strokes = 4572; white matter strokes = 1707; brainstem strokes = 3049; cerebellar strokes = 1951; for *R*_norm_(*Eglob*), *F*(5,25169) = 13.71, *P* < 0.001; for *R*_norm_(modularity), *F*(5,25169) = 42.08, *P* < 0.001]. ** *P* < 0.01, *** *P* < 0.001. Error bars correspond to the standard error of the mean.

Primary stroke sites were categorized into five locations: subcortical (*n* = 13), cortical (*n* = 27), cortico-subcortical (*n* = 15), white matter only (*n* = 5), brainstem (*n* = 9), and cerebellum (*n* = 6). Resilience to virtual attacks was associated with primary stroke location (*P* < 0.001 for both global efficiency and modularity) (Table 2). Highest resilience was observed in cortico-subcortical primary lesions. Specifically, *R*_norm_(*E*_glob_) pooled across all three TPs was 0.31 ± 0.04 for cortico-subcortical primary lesions compared to 0.10 ± 0.02 for other locations. Similarly, *R*_norm_(modularity) pooled across all three TPs was 1.54 ± 0.10 for cortico-subcortical primary lesions compared to 0.48 ± 0.04 for other locations; Fig. 6).

## Discussion

We have investigated resilience of brain networks in a cohort of 75 stroke patients with primary lesions of different sizes and locations. We observed increased network resilience to recurrent virtual lesions in patients compared to controls. Following stroke, patients showed a redistribution of nodal PC, with reduced PC of brain hubs. We further identified patient and primary lesion factors that are associated with brain resilience.

### Increased resilience following stroke

Brain networks exhibit intrinsic robustness, as demonstrated by inherent resilience to targeted and random attacks.^24-27^ We hypothesized that resilience may also represent an adaptation mechanism, triggered by diverse stimuli, including acute strokes. To test this, we simulated recurrent strokes by removing clinically relevant nodes from brain connectivity matrices of patients and controls. Our findings reveal that stroke patients’ brain networks were more resistant to virtual lesions than controls. While we cannot infer on the resulting clinical impact, greater global efficiency and modularity of brain networks should likely translate into better function. We have previously demonstrated that resilience may build up in highly selected stroke patients affected by a first-ever stroke within the primary motor cortex.^29^ However, this population was not representative of the large variability of stroke patterns encountered in clinical practice. In this study, clinical stroke masks were used to simulate secondary lesions, offering a scenario more representative of real-world clinical settings than targeted or random node deletion.

### Architectural specificities of stroke patient networks that may sustain resilience

We next investigated specificities of brain network architecture that may sustain increased resilience following a first stroke, focusing on nodal PC redistribution. Nodes with high PC, or hubs, play a key role in facilitating information flow across modules.^30,47^ Lesions affecting these hub nodes lead to reduced network modularity and widespread cognitive dysfunctions.^22,48^ In this study, we observed a shift in the distribution of nodal PC in patients, compared to controls. Specifically, patients showed lower PC values in these hubs, potentially reducing vulnerability of brain networks to recurrent strokes targeting these critical nodes. Also, the lower the hub PC values in patients, the greater the resilience of their brain networks. Conversely, nodes with low PC in controls had higher values in patients, correlating with increased resilience. These nodes notably consisted in basal ganglia and thalamic nuclei, which are likely engaged in specialized functional loops. While these structures may play central roles within their respective subsystems, they might be less involved in bridging multiple brain modules. By more evenly distributing connections across modules, recurrent events may have a reduced impact on global information flow, thereby preserving global efficiency. Interestingly, a lower mean network PC has been associated with enhanced global efficiency in the developing brain.^31^ Additionally, brain networks of schizophrenic patients exhibit a more random organization, with fewer hubs, contributing to improved resilience to targeted attacks, similar to our findings.^49^

We also evaluated baseline global efficiency, modularity and CCs before simulation of virtual strokes to determine the impact of baseline values on post-attack results. As reported in other studies, global efficiency was significantly reduced in patients within 2 weeks of stroke onset.^23,50,51^ It could be hypothesized that networks with inherently lower global efficiency might artificially exhibit greater resilience to virtual strokes, as new lesions would have a minimal impact on networks that are already severely affected. However, we found no significant correlation between pre-attack global efficiency and resilience, suggesting that baseline graph metrics did not directly affect post-attack values. Additionally, baseline modularity and CC did not differ significantly between patients and controls, despite both metrics showing increased resilience to virtual lesions in patients.

### Patient and primary lesion factors associated with resilience

Finally, we examined patient and primary lesion factors associated with increased resilience. Resilience of global efficiency was higher in haemorrhagic compared to ischaemic strokes, whereas the opposite trend was observed for modularity. Lesion size was not associated with resilience, but primary stroke location had an impact with greater resilience observed in patients with cortico-subcortical primary lesions. Regarding patient-specific factors, female sex was positively associated with resilience of global efficiency, whereas males exhibited greater resilience of modularity. This association aligns with known gender differences in brain organization, where females tend to have more efficient but less segregated brain networks compared to males.^52-55^ Age was positively associated with resilience of network modularity but negatively associated with resilience of network global efficiency. Interestingly, decreased modularity of network connectivity has been liked to aging and is associated with reduced cognitive function.^52,56-58^ In contrast, data on global efficiency are less consistent with some studies reporting decreases and others showing stable in the aging population.^52,53,56,57^ The increased resilience of modularity observed in our study may help preserve a network organization that is already compromised with aging. Finally, more severe clinical deficits, as indicated by higher NIHSS scores, were associated with higher resilience of network global efficiency but not modularity. This relationship does not seem to be driven by larger lesions causing greater clinical deficits and baseline network disruption, which could artificially enhance resilience by limiting the impact of a subsequent lesion, as no correlation was found between lesion size and resilience.

### Limitations

One technical limitation of our study is the relatively large voxel size of functional images, which may lead to partial volume effects that may reduce the accuracy of our analyses. While an atlas-based parcellation was used to mitigate this issue, small regions, such as deep grey matter structures, may still remain susceptible to this effect.

Virtual lesions were used to mimic recurrent brain insults, addressing the challenges of studying real-life stroke recurrence. However, virtual attacks are not equivalent to clinical strokes, which may have distinct effects on neural networks. For instance, ischaemic tissues may retain some function that is not captured in our model. Also, virtual attacks assess immediate changes of neural networks but cannot infer on long-term consequences of clinical lesions. For instance, comparisons of graph metrics derived from chronic stroke patients and virtual lesioning of healthy control connectomes showed differences that suggest long-term network changes that are not captured by virtual attacks.^28^ In our study, virtual lesions involved the removal of cortical and subcortical grey matter nodes from whole-brain connectivity matrices. The impact of recurrent events targeting white matter tracks was not evaluated. New methods developed to investigate fMRI signals of the white matter, along with the newly conceptualized white matter–grey matter functional network may offer valuable opportunities to explore the impact of recurrent strokes affecting white matter tracts.^59,60^ Finally, even though optimization of global efficiency and modularity after a recurrent event could be expected to improve functional outcome, virtual lesions do not allow us to verify this hypothesis.

Further studies might shed light on more specific network changes that support resilience of brain networks, and on their clinical relevance. In particular, neuromodulation after stroke may not only intend to improve the behaviour but also enhance resilience to recurrent strokes.

## Supplementary Material

fcaf218_Supplementary_Data

## Data Availability

Imaging and behaviour data from the Washington stroke cohort are available at https://cnda.wustl.edu/app/template/Login.vm.^32^ Software used for analyses is cited in the ‘Materials and Methods’ section. Code used for this study is available at https://github.com/JulianKlug/stroke-resilience.
