# Supplementary material for: Determinants of brain network resilience after stroke
Source: Brain Commun. 2025 Jun 6;7(3):fcaf218. doi: 10.1093/braincomms/fcaf218 (PMC12198767; doi:10.1093/braincomms/fcaf218)
Supplement: fcaf218_Supplementary_Data [file fcaf218_supplementary_data.pdf]

Supplementary material for

Determinants of brain network resilience after stroke

Supplementary Methods

Graph metrics

As described in the main text, following graph metrics were computed to assess resilience of brain networks: global efficiency (E_{glob}), modularity, clustering coefficient and nodal participation coefficient.

Global efficiency provides a measure of information transfer across all nodes of the network. It quantifies the extent to which nodes communicate with distant nodes. It is proportional to the inverse of the shortest path length.[1]

$$E_{glob} = \frac{1}{n\,(n-1)} \sum_{i \in N} E_i = \frac{1}{n\,(n-1)} \sum_{i \neq j \in N} \frac{1}{d_{ij}}$$

where E_i is the efficiency of node i. N is the set of all nodes in the network, and n is the number of nodes. (i, j) is a link between nodes i and j, (i, j ∈N). d_{ij} is the shortest path length (distance), between nodes i and j.

Modularity is a measure of the strength of division of a network into modules. Networks with high modularity maximize the number of within-group edges and minimize the number of between-group edges. The 17-network parcellation described by Yeo *et al.*[2] was used as an *a priori* definition of modules. Using an atlas-driven approach allows to ensure reproducibility of module definition, compared to data-driven approaches.[3]

$$\mathrm{Q} = \frac{1}{2m} \sum_{ij} (A_{ij} - \frac{k_i k_j}{2m})\ \delta_{c_i c_j}$$

Where Q is the modularity of the network, with m edges and an adjacency matrix A. The expected number of edges falling between two vertices i and j if the configuration model is equal to $\frac{k_i k_j}{2m}$, where k_i is the degree of node i. c_i and c_j are the module assignments of nodes i

and j respectively. $\delta_{c_i c_j}$ *is the Kronecker delta function, which equals 1 if* c_i *equals c , and 0 otherwise.*

Whole brain average clustering coefficient was evaluated as another measure of network segregation, as a complement to modularity. The clustering coefficient (CC) is a measure of how well nodes are grouped together in a graph.[4] For a given node it represents the fraction of node neighbors that are neighbors of each other. The clustering coefficient thus ranges from 0 if none of the node neighbors link to each other to 1 if the neighbors form a complete graph, and all link to each other.

$$CC_i = \frac{2L_i}{k_i(k_i - 1)} \qquad (3)$$

Where CC_i *is the clustering coefficient of the node i,* L_i *represents the number of links between the neighbors of node i, and* k_i *is the degree of node i.*

Whole brain clustering coefficient was derived by averaging individual nodes clustering coefficients.

We finally calculated nodal participation coefficient (PC) of patients and controls' baseline connectivity matrices. At the nodal level, participation coefficient captures how evenly distributed a node's connections are across modules.[5] Nodes with participation coefficients closer to 1 have more between module edges and can be considered as hubs for their respective module.[6,7]

$$PC_i = 1 - \sum_s^N \left(\frac{k_{is}}{k_i}\right)^2$$

Where PC_i *is the participation coefficient of the node i, N the number of modules,* k_{is} *is the number of links of node i to nodes in module s, and* k_i *is the degree of node i.*

The brain connectivity toolbox[8] was used to derive graph metrics.

Supplementary Tables

Supplementary Table 1 Brainnetome regions with highest participation coefficients.

Lobe	Gyrus	Label	MNI coordinates
Frontal lobe	Precentral gyrus right	56	33, -7, 57
Parietal lobe	Precuneus right	152	16, -64, 25
Parietal lobe	Precuneus left	149	-8, -47, 57
Frontal lobe	Superior frontal gyrus right	2	7, 16, 54
Frontal lobe	Paracentral lobule left	65	-8, -38, 58
Frontal lobe	Superior frontal gyrus right	1	-5 ,15, 54
Occipital lobe	Lateral occipital cortex left	209	-22, -77, 36
Frontal lobe	Precentral gyrus right	64	51, 7, 30
Limbic lobe	Cingulate gyrus right	184	4, 6, 38
Occipital lobe	Lateral occipital cortex left	201	-46, -74, 3

Supplementary Table 2 Brainnetome regions with lowest participation coefficient.

Lobe	Gyrus	Label	MNI coordinates
Subcortical nucli	Thalamus left	237	-7, -14, 7
Subcortical nucli	Basal ganglia left	225	-23, 7, -4
Subcortical nucli	Basal ganglia right	226	22, 8, -1
Temporal lobe	Parahippocampal gyrus right	116	19, -10, -30
Subcortical nucli	Basal ganglia right	220	15, 14, -2
Temporal lobe	Inferior temporal gyrus	101	-55, -31, -27
Temporal lobe	Parahippocampal gyrus right	110	28, -8, -33
Subcortical nucli	Thalamus right	232	7, -11, 6
Temporal lobe	Parahippocampal gyrus left	115	-19, -12, -30
Subcortical nucli	Thalamus right	231	-7, -12, 5

References

1. Latora V, Marchiori M. Efficient behavior of small-world networks. *Phys Rev Lett*. Nov 5 2001;87(19):198701. doi:10.1103/PhysRevLett.87.198701

2. Yeo BT, Krienen FM, Sepulcre J*, et al*. The organization of the human cerebral cortex estimated by intrinsic functional connectivity. *J Neurophysiol*. Sep 2011;106(3):1125-65. doi:10.1152/jn.00338.2011

3. Siegel JS, Seitzman BA, Ramsey LE*, et al*. Re-emergence of modular brain networks in stroke recovery. *Cortex*. Apr 2018;101:44-59. doi:10.1016/j.cortex.2017.12.019

4. Watts DJ, Strogatz SH. Collective dynamics of 'small-world' networks. *Nature*. Jun 4 1998;393(6684):440-2. doi:10.1038/30918

5. Guimera R, Nunes Amaral LA. Functional cartography of complex metabolic networks. *Nature*. Feb 24 2005;433(7028):895-900. doi:10.1038/nature03288

6. Power JD, Schlaggar BL, Lessov-Schlaggar CN, Petersen SE. Evidence for hubs in human functional brain networks. *Neuron*. Aug 21 2013;79(4):798-813. doi:10.1016/j.neuron.2013.07.035

7. Smith DM, Kraus BT, Dworetsky A, Gordon EM, Gratton C. Brain hubs defined in the group do not overlap with regions of high inter-individual variability. *Neuroimage*. Aug 15 2023;277:120195. doi:10.1016/j.neuroimage.2023.120195

8. Rubinov M, Sporns O. Complex network measures of brain connectivity: uses and interpretations. *Neuroimage*. Sep 2010;52(3):1059-69. doi:10.1016/j.neuroimage.2009.10.003
